# Polycystic Ovary Syndrome (PCOS): A Cross-Sectional Observational Study Analyzing the Quality of Content on YouTube

**DOI:** 10.7759/cureus.45354

**Published:** 2023-09-16

**Authors:** Shereece Clarke, Gurusha Jangid, Summer Nasr, Axelle Atchade, Britney L Moody, Gaurang Narayan

**Affiliations:** 1 Department of Obstetrics and Gynecology, University of the West Indies, Montego Bay, JAM; 2 Department of Obstetrics and Gynecology, Dr. Sampurnanand Medical College, Jodhpur, IND; 3 Department of Medicine, St. George's University, True Blue, GRD; 4 Department of Obstetrics and Gynecology, Indira Gandhi Government Medical College and Hospital, Nagpur, IND

**Keywords:** youtube, vpi, polycystic ovary syndrome (pcos), gqs score, discern score

## Abstract

Introduction: Polycystic ovary syndrome (PCOS), a chronic multifactorial disorder in women of reproductive age group, is a major public health problem. With most women resorting to platforms like "YouTube" that form a perfect source of edutainment, our aim was to analyze the quality of content available regarding the same.

Aims: The aims and objectives of this study were to assess the quality and reliability of content related to PCOS on YouTube by analyzing the DISCERN score, global quality score (GQS), and video power index (VPI).

Methodology: It was a facility-based cross-sectional study undertaken on a single day with each author reviewing 10 videos from YouTube on PCOS using predetermined keywords. The number of likes, dislikes, views, comments, and uploader backgrounds were evaluated. DISCERN score, GQS, and VPI were also calculated for each video. While data entry was done using Microsoft Excel 2020 (Microsoft Corporation, Washington, United States), the analysis was carried out using SPSS Statistics version 16 (SPSS Inc. Released 2007. SPSS for Windows, Version 16.0. Chicago, SPSS Inc.). Categorical variables were expressed as frequency and percentages, and statistical significance was determined using the Kruskal-Wallis test/one-way ANOVA.

Results: A total of 80 videos that fit the inclusion criteria were analyzed. A majority of the videos (80%) were posted a year back with no updates. Only 28.8% of the video content was posted by doctors. Though most videos (96.25%) shared information pertaining to symptomatology, only 45% spoke regarding prevention. Promotional content was noted in 28.75% of the video content. GQS and VPI were better with information being provided by doctors, hospitals, and healthcare organizations (p-value 0.033 and 0.006, respectively).

Conclusions: With women reaching out to edutainment platforms like YouTube to clarify their concerns surrounding lifestyle diseases such as PCOS in the digital era, it becomes relevant to evaluate the quality of content available on such platforms. The findings of the study form a prototype for addressing the existing gaps in the knowledge available on YouTube. Furthermore, the findings warrant frequent monitoring of such available web-based content and delivery of such content only from qualified wellness experts.

## Introduction

Polycystic ovary syndrome (PCOS) is an endocrinopathy that primarily affects women of childbearing age and is considered the most prevalent endocrine disorder [1]. The condition is characterized by a hormonal imbalance that leads to hyperandrogenism and the development of ovarian cysts, which results in symptoms such as chronic anovulation, excessive hair growth, and acne [2,3]. Additionally, PCOS can affect fertility and increase the risk of high-risk pregnancies. Due to the complex interplay of hormones in the body, PCOS can present differently depending on a person's age and other health conditions [1,2]. Patients with PCOS are also at risk of developing type 2 diabetes mellitus as a result of insulin resistance and adipose retention, which carries additional health risks [3]. Consequently, the complexity and associated complications of this condition can evidently be an overwhelming diagnosis for patients to receive.

Healthcare professionals continue to make new discoveries on the complex pathophysiology of PCOS [1]. It is, therefore, essential for patients to have an avenue for acquiring accurate information on managing their condition in their day-to-day lives, whether it’s connecting with people who have similar experiences or getting easy-to-access information about treatment options. Though patients will likely discuss their diagnosis with their doctor, it does not deter them from looking to the Internet for their own research. Additionally, informed patients allow healthcare professionals to provide good patient-centered care and allow proper shared-decision making.

With YouTube being one of the most accessed sites, it is a platform for over 100 million videos providing information on various medical conditions [4]. The platform has become especially popular among the younger population, which is a significant demographic for PCOS given that it primarily affects women of childbearing age [4].

YouTube’s unique method of delivering information through engaging visuals can make it a valuable tool to expand on a patient’s understanding of their medical condition. However, the validity and accuracy of the advice being offered may be a concern. Expanding into this area as a medical professional or healthcare organization can help patients get easy access to the proper information.

The aims and objectives of this study were to assess the quality and reliability of content related to PCOS on YouTube by analyzing the DISCERN score, global quality score (GQS), and video power index (VPI) to identify knowledge gaps, improve patient education, and keep a record of high-quality content to facilitate patient education.

## Materials and methods

This is a cross-sectional type of observational study conducted in April 2023. Six independent authors were tasked with searching and reviewing 10 or more videos from YouTube.com on PCOS using predetermined keywords. Each video was sorted by relevance to the searched keywords: “PCOS,” “polycystic ovarian disease,” “PCOS treatment,” “PCOS prevention,” “PCOS cause,” or “PCOS diet.”

Each author completed a Google form (Google LLC, California, United States) questionnaire after viewing a video. A total of 95 videos were evaluated. A total of 15 videos were excluded based on repeated entries or the failure to uphold the following inclusion criteria: relevance to the topic or subject PCOS, in the “English” language, and video within the length of one minute to 20 minutes.

A total of 80 videos were included in the study and analyzed statistically. Evaluations on the number of likes, dislikes, views, and comments were recorded. The VPI score was calculated with the formula [likes/ (likes + dislikes)] x 100 [5]. The DISCERN score (also known as the reliability score) and the GQS score were calculated independently by each researcher, and their median was included in the statistics. The DISCERN score is a scoring system based on the quality criteria for consumer information, where 1 point asks if the aims are clear, and 5 points asks if all areas of uncertainty are stated [6]. The GQS score scale ranges from 1 (poor quality) to 5 (excellent quality) [7].

Data entry was done using Microsoft Excel 2020 (Microsoft Corporation, Washington, United States), and the analysis was carried out using SPSS Statistics version 16 (SPSS Inc. Released 2007. SPSS for Windows, Version 16.0. Chicago, SPSS Inc.). Categorical variables were expressed as frequency and percentages, and statistical significance was determined using the Kruskal-Wallis test/one-way ANOVA.

## Results

A total number of 95 YouTube videos were evaluated for this study. However, after applying the inclusion/exclusion criteria and after deleting repeated videos, a total of 80 videos were deemed eligible.

Table 1 highlights the characteristics of the YouTube videos that were analyzed. The total number of views was 36,437,534, the total number of likes was 794,779, and the total number of comments was 54,162.

**Table 1:** Characteristics of YouTube videos analyzed

	Absolute number of videos	Percentage (%)
Time since uploaded		
Less than six months ago (<180 days old)	11	13.8
More than six months to one year (>365 days)	5	6.3
More than one year (<365 days)	64	80
Popularity		
Total no. of views	36,437,534	
Total no. of likes	794,779	
Total no. of dislikes	13,044	
Total no. of comments	54,162	
Type of uploader		
Doctor	23	28.8
Hospital	13	16.3
Healthcare organization	4	5
News channel	3	3.8
Patient	7	8.8
Other	30	37.5

Eighty percent of the videos were uploaded after a year with no updates, with 37% being uploaded by members of the "other" category, outnumbering those uploaded by doctors (28.8%), hospitals (16.3%), and healthcare organizations (5%).

Table 2 demonstrates the nature of the information uploaded on these YouTube videos, with 96.25 % of them describing symptoms of PCOS and 77.5% about the cause or etiology. However, only 45.25% discussed the relevant investigations or tests to be done, and only 40% about rehabilitation. About 76.25% of videos discussed information regarding treatment options. Information about support groups and patient-sharing experiences with family members was the least discussed in the videos with both having a cumulative score of 6.25%. About 28.75% of these videos conveyed promotional content by doctors and pharmaceutical companies.

**Table 2:** Information on PCOS by the YouTube videos

Type of information	Absolute number of videos	Percentage (%)
Description of symptoms	77	96.2
Information about cause/etiology	62	77.5
Info about investigations/tests	37	46.2
Info about prevention/vaccines	36	45.0
Info about treatment	61	76.2
Info about mortality	20	25.0
Info about rehabilitation	32	40.0
Info about support groups	3	3.7
Info about people/patients sharing their own experience	11	13.7
Info about patient sharing their experience with their family members	2	2.5
Does the post have promotional content by pharmaceutical companies or doctors?	23	28.7

Comparisons of GQS, reliability score, and VPI were made based on the type of uploader as shown in Table 3. The Kruskal-Wallis test was used to analyze the medians for each category. The VPI and GQS scores had a significant difference among the types of uploaders, with p-values of 0.006 and 0.003, respectively. This indicates that doctors, hospitals, and healthcare organizations were more likely to offer more quality content in their videos compared to other categories. However, the reliability score showed no significant difference among groups, with a p-value of 0.07.

**Table 3:** Comparison of GQS, reliability score, and VPI based on the type of uploader The data is presented in the format: median (IQ1, IQ3), where IQ stands for quartile. p<0.05 is significant. GQS: global quality score, VPI: video power index

Measures of comparison	Doctors (n=23)	Hospitals (n=13)	Healthcare organization (n=4)	News agency (n=3)	Patient (n=7)	Others (n=30)	p-value and test used
	Median (IQ1, IQ3)	Median (IQ1, IQ3)	Median (IQ1, IQ3)	Median (IQ1, IQ3)	Median (IQ1, IQ3)	Median (IQ1, IQ3)	Kruskel-Wallis test
VPI	140.54 (7.54, 502.14)	5.15 (0.9, 121.44)	649.01 (201.21, 1428.38)	12.7 (12.69, )	36.27 (8.69, 413.85)	374.04 (27.36, 945.85)	p-value = 0.006
GQS	4 (3, 4)	4 (4, 5)	4 (3.25, 4)	3 (2, )	3 (2, 3)	4 (3, 4)	p-value = 0.033
Reliability score	4 (3, 4)	4 (3.25, 4.5)	4 (3.25, 4)	2 (1, )	3 (1, 3)	3.5 (3, 4)	p-value = 0.070

## Discussion

PCOS is an endocrine disorder that mostly affects women of reproductive age. The proportion of women with PCOS has increased in the last decade [8]. Similarly, Internet searches for PCOS have increased over time, especially when compared to those for other common gynecological illnesses [9]. Most patients consider online health resources to be on par with or even superior to those supplied by their doctors, and many of those who consult the web for medical advice do not share the findings of their searches with their doctors [10]. In the reproductive female population, PCOS is a common endocrine condition. These young females can readily and frequently view YouTube videos online to learn more about their illnesses and discover possible treatments [11].

The content, providers, and quality of these videos vary markedly. Our study has found that YouTube videos about PCOS have a large number of views, thus having a major influence on the viewers. The total views on the evaluated videos were 36,437,534. The majority of the videos analyzed were uploaded by non-physicians followed by videos uploaded by doctors (28.8%) and hospitals (16.3%).

Hyperandrogenism (clinical or biochemical) and irregular or absent menstrual periods (oligomenorrhea or amenorrhea) with chronic anovulation are the hallmark features of PCOS [12]. Our study highlighted that symptoms were described in nearly 96.25% of videos, while only 3.75% of videos had more information about support groups.

In addition to the complexities inherent in the multifactorial physiopathology of PCOS, the diagnosis can be difficult to make for two reasons: first, there are a variety of diagnostic criteria to choose from, and second, there are debates about how to define and assess the key symptoms of PCOS [13]. Delayed diagnosis and dissatisfaction with care along with gaps in management have been the major concerns for women across the globe [14]. Our study found that only 46.25% of examined videos contained information about investigations and diagnostic tests, but 76.25% of the videos under study had information about the treatment of PCOS. This ascertains the challenges and lack of reliable information about the diagnosis and management of PCOS.

Multi-component healthy lifestyle treatments are the primary therapeutic alternatives for treating PCOS and avoiding associated comorbidities [14]. Approximately 45% of the YouTube videos evaluated and discussed prevention. A notable 28.75% of videos had promotional content by pharmaceutical companies or doctors.

Various indices are mentioned in the literature to evaluate the quality of videos uploaded online [15]. In our study, we used VPI, GQS, and DISCERN scores to evaluate the examined videos. The median GQS of videos uploaded by doctors, hospitals, and healthcare organizations were slightly more than those uploaded by patients and news agencies. Findings are consistent with other studies wherein informational videos by doctors and hospitals had better quality [16]. It is evident that better information and knowledge about the disease and its management correlates with higher quality.

There was a statistically significant difference between the median VPI of different uploaders. The median VPI was highest for videos uploaded by healthcare organizations followed by doctors and then the news agency. Informational videos by hospitals had the lowest median VPI despite having a high GQS and DISCERN score. Videos uploaded by doctors, hospitals, and healthcare organizations had a higher median DISCERN score of 4 each when compared to those of news agencies and patients. This further goes on to explain the reliability of content by professional healthcare providers.

Videos uploaded by patients also had a higher popularity in terms of VPI than videos uploaded by hospitals. Although videos uploaded by patients had a lower GQS when compared to the GQS of hospital uploaders. This finding is similar to some of the earlier studies like that of Loeb et al. on “Dissemination of Misinformative and Biased Information about Prostate Cancer on YouTube,” where videos uploaded by patients had higher engagement [17].

There were several limitations in our study. First, the number of YouTube videos selected was less, compared to the vast number of videos available on the Internet. We only selected the videos that were trending and easily visible to the viewers. Second, we did not study the effect of promotional videos by pharmaceutical companies on the type of content. This study was aimed at finding the type of information available on YouTube about PCOS.

## Conclusions

In our study, the quality and reliability of videos on PCOS published on YouTube were low to average. However, uploaders like doctors, hospitals, and healthcare organizations had higher quality content than others. This was reflected in higher GQS and DISCERN scores. However, a notable percentage of videos also contained promotional content by doctors and pharmaceutical companies.

A smaller number of videos had information about diagnosis and treatment when compared to videos discussing symptoms. We hope that our study will help shed light on the diagnosis, treatments, and prevention of PCOS and the dissemination of information about the same on widely used social media platforms like YouTube.

There is a need for healthcare organization groups and government agencies to join hands and develop methodologies for the proper and regular screening of healthcare-related videos posted on various social media websites like YouTube. This will ensure that high-quality content with reliable information is circulated among the general population that not only gives them correct information about common healthcare conditions like PCOS but also disperses myths and misinformation. Further studies should be directed toward developing and implementing such methodologies.
